# Regulation of the Expression of the *Vibrio parahaemolyticus peuA* Gene Encoding an Alternative Ferric Enterobactin Receptor

**DOI:** 10.1371/journal.pone.0105749

**Published:** 2014-08-22

**Authors:** Tomotaka Tanabe, Ayaka Kato, Keiichi Shiuchi, Katsushiro Miyamoto, Hiroshi Tsujibo, Jun Maki, Shigeo Yamamoto, Tatsuya Funahashi

**Affiliations:** 1 Laboratory of Hygienic Chemistry, College of Pharmaceutical Sciences, Matsuyama University, Matsuyama, Ehime, Japan; 2 Graduate School of Medicine, Dentistry and Pharmaceutical Sciences, Okayama University, Okayama, Japan; 3 Department of Microbiology, Osaka University of Pharmaceutical Sciences, Takatsuki, Osaka, Japan; 4 Laboratory of Infectious Diseases, College of Pharmaceutical Sciences, Matsuyama University, Matsuyama, Ehime, Japan; State Key Laboratory of Pathogen and Biosecurity, Beijing Institute of Microbiology and Epidemiology, China

## Abstract

A *pvsB*-*vctA*-*irgA* triple deletion mutant of *Vibrio parahaemolyticus* can utilize enterobactin under iron-limiting conditions by inducing a previously undescribed receptor, PeuA (VPA0150), in response to extracellular alkaline pH and enterobactin. *In silico* analyses revealed the existence of a two-component regulatory system operon, *peuRS*, immediately upstream of *peuA*, which constitutes an operon with the TonB2 system genes. Both the *peuRS* and *peuA-tonB2* operons were found to be upregulated under iron-limiting conditions in a ferric uptake regulator (Fur)-dependent manner. The involvement of *peuA* and *peuRS* in enterobactin utilization was analyzed by complementation experiments using deletion mutants. Primer extension analysis indicated that, under iron-limiting conditions, the transcription of *peuA* was initiated from the +1 site at pH 7.0 and from both the +1 and +39 sites at pH 8.0 in the presence of enterobactin. The +39 transcript was absent from the *peuRS* deletion mutant. Secondary structure prediction of their 5′-untranslated regions suggested that translation initiation is blocked in the +1 transcript, but not in the +39 transcript. Consistent with this, *in vitro* translation analysis demonstrated that production of PeuA was determined only by the +39 transcript. These studies establish a novel gene regulation mechanism in which the two-component regulatory system PeuRS enhances expression of the alternative +39 transcript that possesses non-inhibitory structure, allowing the *peuA* expression to be regulated at the translation stage.

## Introduction

Iron is essential for the growth of nearly all forms of life, but its very limited solubility makes iron scarce. The predominant type (ferric iron) preferentially forms barely soluble hydroxide complexes under aerobic conditions and at neutral and alkaline pH [1]–[3]. To solubilize iron from these complexes and to acquire adequate levels, bacteria as well as other microorganisms frequently secrete siderophores, including catecholates, hydroxamates, and polycarboxylates [2], [4], all of which exhibit high affinity for ferric iron. In Gram-negative bacteria, ferric siderophore complexes thus formed in the extracellular milieu are conveyed into the bacterial cell by a high-affinity active transport system composed of an outer membrane receptor (OMR) coupled with both a TonB-ExbBD protein complex (known as a TonB system) and an ATP-binding cassette (ABC) transporter system [5], [6]. The TonB system transduces the proton-motive force of the cytoplasmic membrane to the OMRs [5], [6], which are therefore known as TonB-dependent receptors. It is well known that, in these processes, the siderophore specificity resides mainly in the OMRs [2], [7]. Furthermore, expression of the genes responsible for iron acquisition is regulated by the cellular pool of iron through a ferric uptake regulator (Fur), which is ubiquitous in Gram-negative bacteria and usually acts as a repressor with ferrous iron as a co-repressor [8], [9]. When the intracellular iron concentration increases, the Fur-Fe^2+^ complex binds to a consensus sequence, termed the Fur box, located in the promoter regions of the Fur target genes, thereby leading to repression of transcription initiation. In contrast, when iron becomes scarce in the cell, Fur is inactivated by release of the iron cofactor, and the target genes are transcribed to efficiently scavenge iron from the surroundings.

In addition to their own siderophores, some bacteria have evolved transport systems for ferric complex that use exogenous siderophores (xenosiderophores) produced by other bacterial or fungal species [10]. This strategy, called siderophore piracy [11], may be highly advantageous for survival and proliferation of these bacteria, because it allows them to escape any bacteriostatic or competitive effects caused by xenosiderophores likely to coexist under various environmental conditions [12]. In *Pseudomonas aeruginosa*, the ferric enterobactin (Ent) receptor PfeA is induced in the presence of Ent combined with iron-starvation via the PfeRS two-component regulatory system [13], [14]. This system typically comprises an inner membrane-integrated histidine sensor kinase and a cytoplasmic response regulator that together form a signal transduction pathway to regulate gene expression; environmental stimuli, including a wide range of physical and chemical signals, trigger autophosphorylation of a histidine sensor kinase, and its phosphoryl group is subsequently transferred to a response regulator, which activates or represses transcription of the target gene required for the appropriate physiological response [15]–[17].

*Vibrio parahaemolyticus* is a Gram-negative and halophilic human pathogen that naturally inhabits marine and estuarine environments. It is a significant cause of acute gastroenteritis worldwide, acquired through the consumption of raw or undercooked seafood [18]–[20]. Under iron-limiting conditions, this bacterium secretes its own siderophore, vibrioferrin [21], which is biosynthesized by four enzymes encoded by *pvsABDE* [22], and transports extracellular iron as ferric vibrioferrin back to the cell via two OMRs specific to ferric vibrioferrin and the ABC transporter complex, which are encoded by *pvuA1-pvuA2* and *pvuBCDE*, respectively [22]–[24]. In addition to producing vibrioferrin, *V. parahaemolyticus* can utilize aerobactin [25], ferrichrome [26], and Ent [27] as xenosiderophores by expressing their cognate OMRs.

In this report, we show that the *V. parahaemolyticus peuA* gene encoding the ferric Ent receptor is responsible for Ent utilization under iron-limiting conditions at pH 8.0. We also present evidence that the expression of PeuA is determined by an alternative transcript (+39 transcript) of *peuA* that is induced under iron-limiting conditions via a two-component regulatory system encoded by *peuRS* in response to extracellular alkaline pH and Ent.

## Materials and Methods

### Bacterial Strains, Plasmids, Growth Conditions, and Primers

The bacterial strains and plasmids used in this study are listed in Table 1 and S1, respectively. *Escherichia coli* β2155 [29], which is a diaminopimelic acid auxotroph, was grown under routine conditions and maintained in Luria-Bertani (LB) medium containing 0.5% NaCl and 0.5 mM 2,6-diaminopimelic acid. *V. parahaemolyticus* RIMD2210633 [28] and its deletion mutants were incubated in LB medium containing 3% NaCl or in LB medium containing 3% NaCl and 100 mM Tris-HCl (LB-Tris medium) at pH 7.0 and 8.0. To impose iron limitation on *V. parahaemolyticus* strains, they were grown in LB-Tris medium containing 25 µM ethylenediamine-di(*o*-hydroxyphenylacetic acid) (EDDA; Sigma-Aldrich) (LB-Tris/+EDDA medium). When required, the siderophore Ent (Sigma-Aldrich) was added to the LB-Tris/+EDDA medium at a final concentration of 5 µM (LB-Tris/+EDDA/+Ent medium). Antibiotics were added at the following concentrations: 10 µg/mL chloramphenicol and 10 µg/mL tetracycline. The oligonucleotide primers used in this study are listed in Table S2.

**Table 1:** Bacterial strains used in this study.

Strain	Description	Reference or source
*V. parahaemolyticus*		
RIMD2210633	Clinical isolate of serotype O3:K6; wild-type strain	[28]
VPD5	RIMD2210633 Δ*pvsB* (vibrioferrin-deficient mutant)	[24]
VPD54	VPD5 Δ*vctA* Δ*irgA*	[27]
VPD55	VPD5 Δ*vctA* Δ*peuA*	This study
VPD56	VPD5 Δ*irgA* Δ*peuA*	This study
VPD57	VPD5 Δ*vctA* Δ*irgA* Δ*peuA*	This study
VPD72	VPD5 Δ*vctA* Δ*irgA* Δ*tonB1*	This study
VPD73	VPD5 Δ*vctA* Δ*irgA* Δ*tonB2*	This study
VPD74	VPD5 Δ*vctA* Δ*irgA* Δ*tonB3*	This study
VPD102	VPD5 Δ*vctA* Δ*irgA* Δ*peuRS*	This study
VPD107	VPD5 Δ*pvuA1* Δ*pvuA2* Δ*hutA* Δ*fhuA* Δ*iutA* Δ*vctA* Δ*irgA*	This study
VPD108	VPD5 Δ*pvuA1* Δ*pvuA2* Δ*hutA* Δ*fhuA* Δ*iutA* Δ*vctA* Δ*irgA* Δ*peuA*	This study
VPD109	VPD5 Δ*pvuA1* Δ*pvuA2* Δ*hutA* Δ*fhuA* Δ*iutA* Δ*vctA* Δ*irgA* Δ*peuRS*	This study
VPD110	VPD5 Δ*vctA* Δ*irgA* Δ*VP0168*	This study
*E. coli*		
β2155	*thrB1004 pro thi strA hsdS* Δ(*lacZ*)ΔM15 (F′ Δ(*lacZ*)M15 *lacI* ^q^ *traD36 proA* ^+^ *proB* ^+^) Δ*dapA*::*erm*(Em^r^), *pir*::RP4(::*kan*(Km^r^) from SM10)	[29]

### Growth Assay

The growth assay was performed using a TVS062CA biophotorecorder (Advantec Toyo, Tokyo, Japan). Briefly, *V. parahaemolyticus* cells grown overnight in LB medium were diluted with LB-Tris/+EDDA or LB-Tris/+EDDA/+Ent medium to an optical density at 600 nm (OD_600_) of 0.005. The cultures were shaken at 70 rpm at 37°C, and the OD_600_ was measured every 3 h for 18 h.

### DNA Manipulation and *in silico* Sequence Analysis

Chromosomal DNA was extracted with a Wizard genomic DNA purification kit (Promega), and plasmid DNA was routinely prepared with a High Pure Plasmid Isolation Kit (Roche), according to the manufacturer's instructions. Standard DNA manipulation was performed as described [30]. Homology searches were performed using the BLAST program of the National Center for Biotechnology Information (http://blast.ncbi.nlm.nih.gov/) [31].

### Preparation of Outer Membrane Protein (OMP)-Rich Fractions and Sodium Dodecyl Sulfate-Polyacrylamide Gel Electrophoresis (SDS-PAGE)

Stationary-phase *V. parahaemolyticus* cells were inoculated at a final OD_600_ of 0.005–0.01 into LB-Tris, LB-Tris/+EDDA, and LB-Tris/+EDDA/+Ent media at pH 7.0 and 8.0, and the cultures were shaken at 37°C for 4 h. Sarkosyl-insoluble OMPs were prepared and analyzed by SDS-PAGE, as previously described [32]. Separated OMPs were electroblotted onto a wet polyvinylidene difluoride membrane, and the N-terminal amino acid sequence was determined using the Edman degradation method with a Procise 491 HT protein sequencer (Applied Biosystems).

### Gene Deletion and Complementation

Gene deletions in the *V. parahaemolyticus* genome were constructed by allelic exchange using the suicide plasmid pXAC623, according to the procedure described by Kuroda *et al*. [33]. Briefly, DNA fragments with deletions in the *peuA*, *peuRS*, *hutA*, *iutA*, *fhuA*, and *VP0168* genes were prepared by overlap extension PCR [34], as previously described [24]. The deleted gene fragments were ligated into appropriately digested pXAC623 to yield pXAC623ΔpeuA, pXAC623ΔpeuRS, pXAC623ΔhutA, pXAC623ΔiutA, pXAC623ΔfhuA, and pXAC623ΔVP0168 (Table S1), which were then transformed into *E. coli* β2155 to generate the respective donor strains. After filter mating between each donor strain and an appropriate *V. parahaemolyticus* strain, merodiploid recombinants were selected on LB plates containing chloramphenicol, but not diaminopimelic acid. Each merodiploid recombinant was spread on VDS-broth agar plates (1% polypeptone, 0.5% yeast extract, 30 mM NaCl, 55 mM KCl, 10% sucrose, and 2.5% agar) [33] and incubated at 25°C for 30 h, at which point sucrose-resistant and chloramphenicol-sensitive colonies were selected. The deletions were verified by PCR, using chromosomal DNA isolated from each deletion mutant (data not shown). To complement the *peuA* and *peuRS* deletion mutants, PCR amplicons containing the respective genes were ligated into appropriately digested pRK415 [35], and the resulting complementing plasmids were transformed into the respective *V. parahaemolyticus* mutant strains.

### RNA Analysis

Stationary-phase *V. parahaemolyticus* cells were inoculated as described for the preparation of OMP-rich fractions, and the cultures were then shaken at 37°C until they reached OD_600_ 0.3–0.6. Each cell pellet was treated with the RNAprotect Bacteria Reagent (Qiagen), according to the manufacturer's instructions, and total RNA was prepared from each cell sample using the RNeasy Mini kit (Qiagen) or TriPure Reagent (Roche), according to the manufacturer's instructions. Total RNA samples thus obtained were used for primer extension, reverse transcriptase (RT)-PCR, and RT-quantitative (q) PCR.

Primer extension analysis of *peuA* and *peuR* was performed with the oligonucleotide primers peuA-PE and peuR-PE (see Table S2), respectively, which had been 5′-labeled with Texas Red prior to use. Each labeled primer was annealed to 10 µg or 150 µg of total RNA and extended with avian myeloblastosis virus RT XL (TaKaRa Biochemicals, Shiga, Japan) at 50°C for 90 min. The primer extension products were separated on a sequencing gel using an SQ5500E DNA sequencer (Hitachi High-Tech, Tokyo, Japan) alongside the DNA sequence ladder of the control region synthesized using the same primers used for the primer extension analysis.

For RT-PCR analysis, total RNA samples prepared from RIMD2210633 cells grown in LB-Tris/+EDDA medium at pH 7.0 were treated with TURBO DNase (Ambion) to remove contaminating chromosomal DNA. ReverTra Ace RT (Toyobo, Osaka, Japan) and the gene-specific primer VPA0156-R or peuS-R (see Table S2) was used to synthesize cDNA. cDNA synthesis was performed by incubating 0.5 µg of DNase-treated RNA in a 20-µl reaction for 60 min at 42°C. One microliter of the cDNA reaction mixture was then used as a template for PCR with the specific PCR primer pairs (see Table S2). PCR conditions were as follows: after an initial denaturation for 2 min at 95°C, DNA was amplified for 30 cycles, with each cycle consisting of denaturation at 95°C for 30 s, annealing at 55°C for 30 s, and extension at 72°C for 1 min. As a negative control, PCR omitting prior reverse transcription was performed directly for the same RNA template to confirm the absence of contaminating chromosomal DNA. PCR products were electrophoresed through 1.5% agarose gels, stained with ethidium bromide, and visualized with UV light.

For RT-qPCR analysis, total RNA samples were treated with TURBO DNase, and a 0.5-µg aliquot of RNA was reverse transcribed with the ReverTra Ace RT and random hexamer primers (TaKaRa Biochemicals) for 60 min at 37°C. qPCR was performed using the *peuA*-specific primer pair VppeuA-qF/VppeuA-qR (see Table S2) and the Thunderbird SYBR qPCR Mix (Toyobo) in a Chromo4 Real-Time PCR detection system (Bio-Rad) under the conditions specified in the manufacturer's protocol. Relative mRNA expression levels were determined by the comparative threshold cycle method, using the 16S rRNA expression level as an internal control. The RT-qPCR primers for 16S rRNA are listed in Table S2.

### Preparation of DNA Templates and *in vitro* RNA Synthesis

Both a longer form of the truncated *peuA* fragment (+1 to +402) flanked by the T7 promoter and *flag* sequences (+1-*peuA*′-*flag* DNA) and a shorter form of the truncated *peuA* fragment (+39 to +402) flanked by T7 promoter and *flag* sequences (+39-*peuA*′-*flag* DNA) were amplified from *V. parahaemolyticus* chromosomal DNA by PCR. The primer pairs T7-VppeuA-F/T7-VppeuA-FLAG-R and T7-VppeuA-F/T7-VppeuA-FLAG-R (see Table S2) were used to amplify the longer form and the shorter form, respectively. A full-length *fur* fragment flanked by the T7 promoter and *flag* sequences (*fur*-*flag* DNA) was amplified by first-step PCR using *V. parahaemolyticus* chromosomal DNA and the primer pair T7-Vpfur-F/T7-Vpfur-FLAG-R (see Table S2), and subsequently by second-step PCR using the first-step amplicon and the primer pair UNIVERSAL/T7-Vpfur-FLAG-R (see Table S2). The amplified DNA fragments were purified by agarose gel electrophoresis and used as templates for *in vitro* RNA synthesis. The +1-*peuA*′-*flag*, +39-*peuA*′-*flag*, and *fur*-*flag* RNAs were synthesized from the +1-*peuA*′-*flag*, +39-*peuA*′-*flag*, and *fur*-*flag* DNAs, respectively, by *in vitro* transcription with T7 RNA polymerase (Roche). Following the *in vitro* transcription reaction, the reaction mixtures were treated with 1 U RQ1 DNase (Promega) and purified on a ProbeQuant G50 Micro column (GE Healthcare), followed by ethanol precipitation.

### *In vitro* Translation Assay and Western Blotting

*In vitro* translation was performed using a PURESYSTEM classic II (BioComber, Tokyo, Japan). For the RNA template for *in vitro* translation, either a +1-*peuA*′-*flag* RNA (30 pmol)/*fur*-*flag* RNA (3 pmol) mixture or a +39-*peuA*′-*flag* RNA (30 pmol)/*fur*-*flag* RNA (3 pmol) mixture was used. The PURESYSTEM reaction mixture (20 µl) was incubated at 37°C for 2 h, and the reaction was then terminated by adding an equal volume of 2× SDS-PAGE sample buffer. The samples were separated on a 15% SDS-polyacrylamide gel, and the protein bands were transferred to a Clear Blot Membrane-P (Atto, Tokyo, Japan). The membrane was blocked with Tris-buffered saline with Tween 20 (TBST) containing 0.3% skim milk and incubated overnight at 4°C with mouse anti-FLAG M2 antibody (Sigma) diluted 1,000-fold with blocking solution. The membrane was then washed four times with TBST, incubated for 1 h at room temperature with horseradish peroxidase-conjugated anti-mouse secondary antibody (GE Healthcare), diluted 20,000-fold with blocking solution, and washed four times with TBST. Immunoreactive bands were detected with an ECL Select Western Blotting Detection Reagent (GE Healthcare) and visualized with a LAS-3000 gel imager (Fujifilm, Tokyo, Japan)

### Northern Blotting

The reaction mixtures (2.5 µl) obtained after *in vitro* translation of +1-*peuA*′-*flag* and +39-*peuA*′-*flag* RNAs were separated by electrophoresis on a 5% polyacrylamide/8 M urea gel in Tris-borate-EDTA buffer (90 mM Tris, 90 mM boric acid, 2 mM EDTA, pH 8.3), blotted onto a Biodyne B positively charged nylon membrane (Pall Corporation), and fixed to the membrane by baking for 30 min at 80°C. The digoxigenin (DIG)-labeled *peuA* probe was prepared with a primer pair, VppeuA-F/VppeuA-R (see Table S2), internal to the *peuA* gene using a DIG PCR Probe Synthesis Kit (Roche). Hybridization was performed overnight at 65°C, and the hybridized DIG-labeled *peuA* probe was detected using a DIG Luminescent Detection Kit (Roche) with a LAS-3000 image analyzer.

## Results

### Identification of the OMR Gene Responsible for Alkaline pH-Dependent Utilization of Ent in *V. parahaemolyticus*

**Figure 1:**
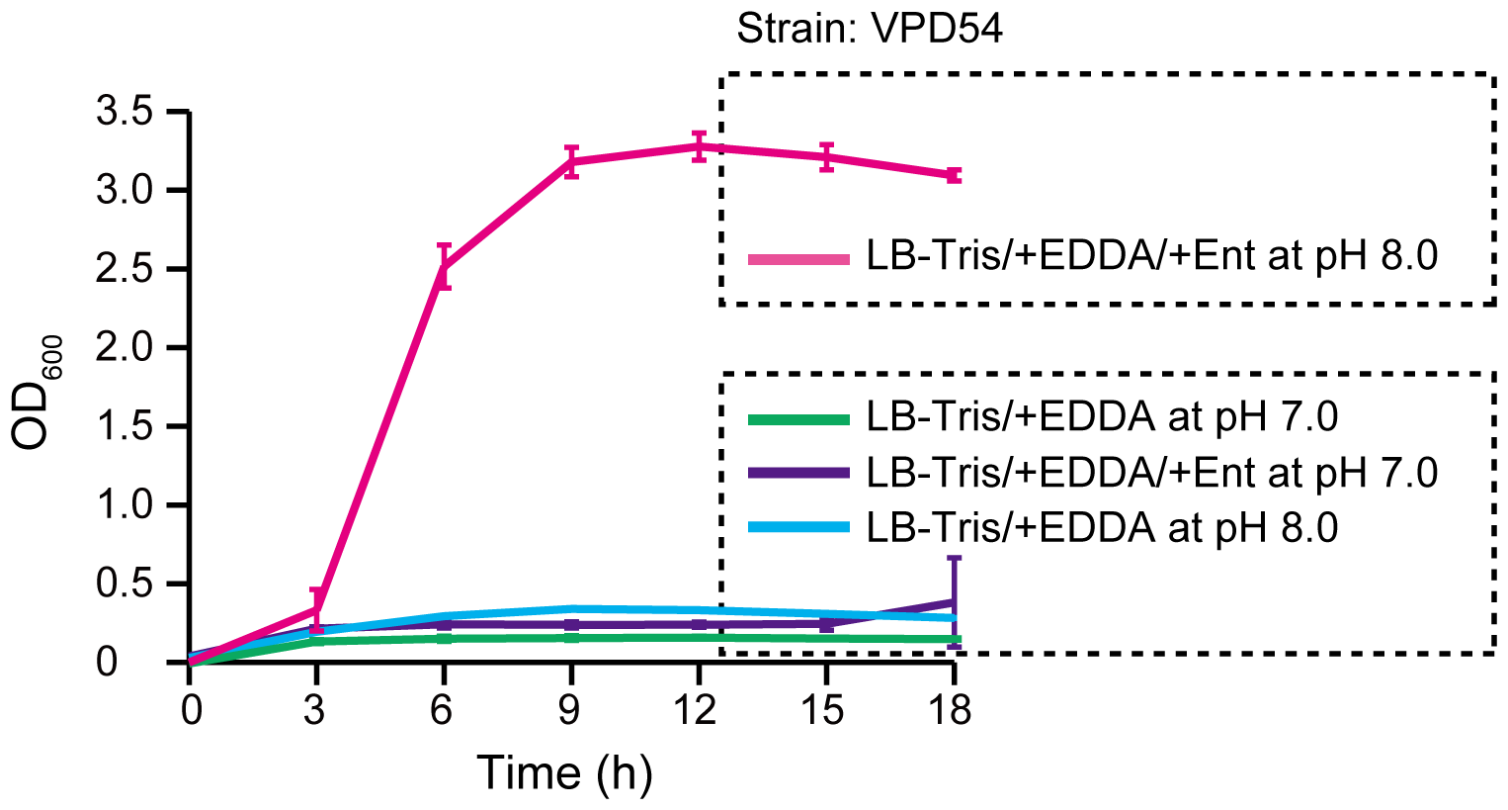
Alkaline pH-dependent utilization of Ent in VPD54. VPD54, which is a *vctA* and *irgA* deletion mutant generated from the VPD5 vibrioferrin-deficient mutant, was grown in LB-Tris/+EDDA medium (at indicated pH) at 37°C for 18 h with shaking at 70 rpm. When required, Ent was added at 5 µM. Cultures were monitored by measuring the OD_600_ every 3 h. Data are shown as means ± SD from 3 separate experiments.

**Figure 2:**
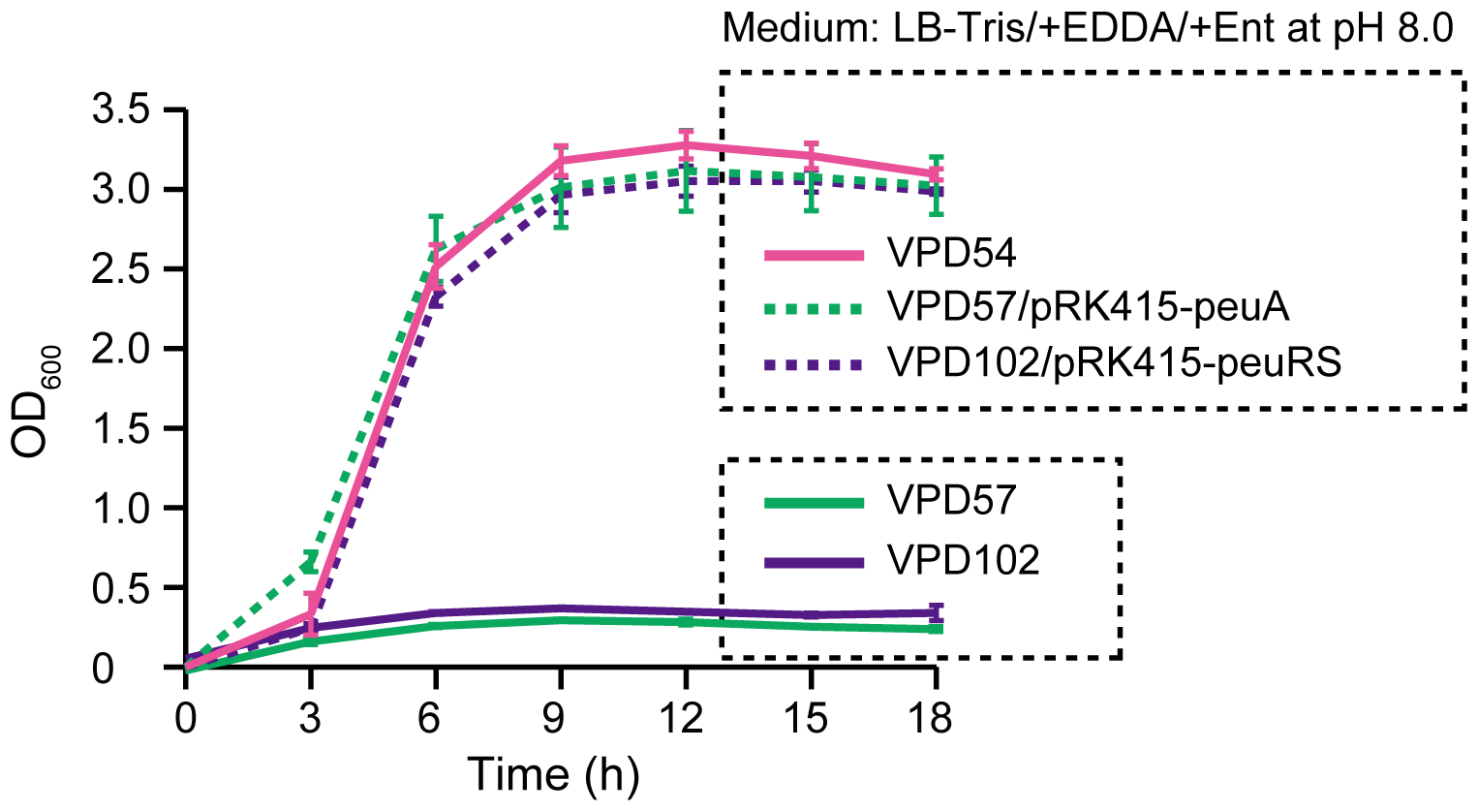
Involvement of *peuA* and *peuRS* in Ent utilization. The growth assay was performed as described in Figure 1. Data are shown as means ± SD from 3 separate experiments.

We previously reported that *V. parahaemolyticus* can utilize ferric Ent as an iron source via the VctA and IrgA receptors [27]. Although the VPD54 mutant with deletion of *vctA* and *irgA* that was generated from the VPD5 vibrioferrin-deficient mutant failed to grow in LB-Tris/+EDDA medium at pH 7.0 and 8.0, it showed normal growth when Ent was added to LB-Tris/+EDDA medium (LB-Tris/+EDDA/+Ent) at pH 8.0, but not at pH 7.0 (Figure 1). These data indicate that *V. parahaemolyticus* possesses a ferric Ent receptor gene that is specifically induced in response to extracellular alkaline pH and Ent. Several ferric siderophore receptors have already been identified and characterized in *V. parahaemolyticus* [23]–[27]; VPA0150 and VP0168 [28] have been documented as putative TonB-dependent receptors, but neither has been proven to be functional. To determine whether these receptors are involved in Ent utilization at pH 8.0, the two genes were deleted from the VPD54 mutant, and the resulting mutants, VPD57 and VPD110, were subjected to a growth assay. VPD110 grew in LB-Tris/+EDDA/+Ent medium at pH 8.0 (data not shown). In contrast, VPD57 showed no growth in LB-Tris/+EDDA/+Ent medium at pH 8.0, and the VPD57/pRK415-peuA complementing strain exhibited growth similar to the wild-type RIMD2210633 in the same medium (Figure 2). These data suggest that the *VPA0150* gene encodes a receptor engaged in the uptake of ferric Ent at pH 8.0. In this paper, the *VPA0150* gene is termed *peuA* (*peu* stands for *V. parahaemolyticus* Ent utilization).

### Identification of PeuA by SDS-PAGE and Determination of Its N-Terminal Amino Acid Sequence

**Figure 3:**
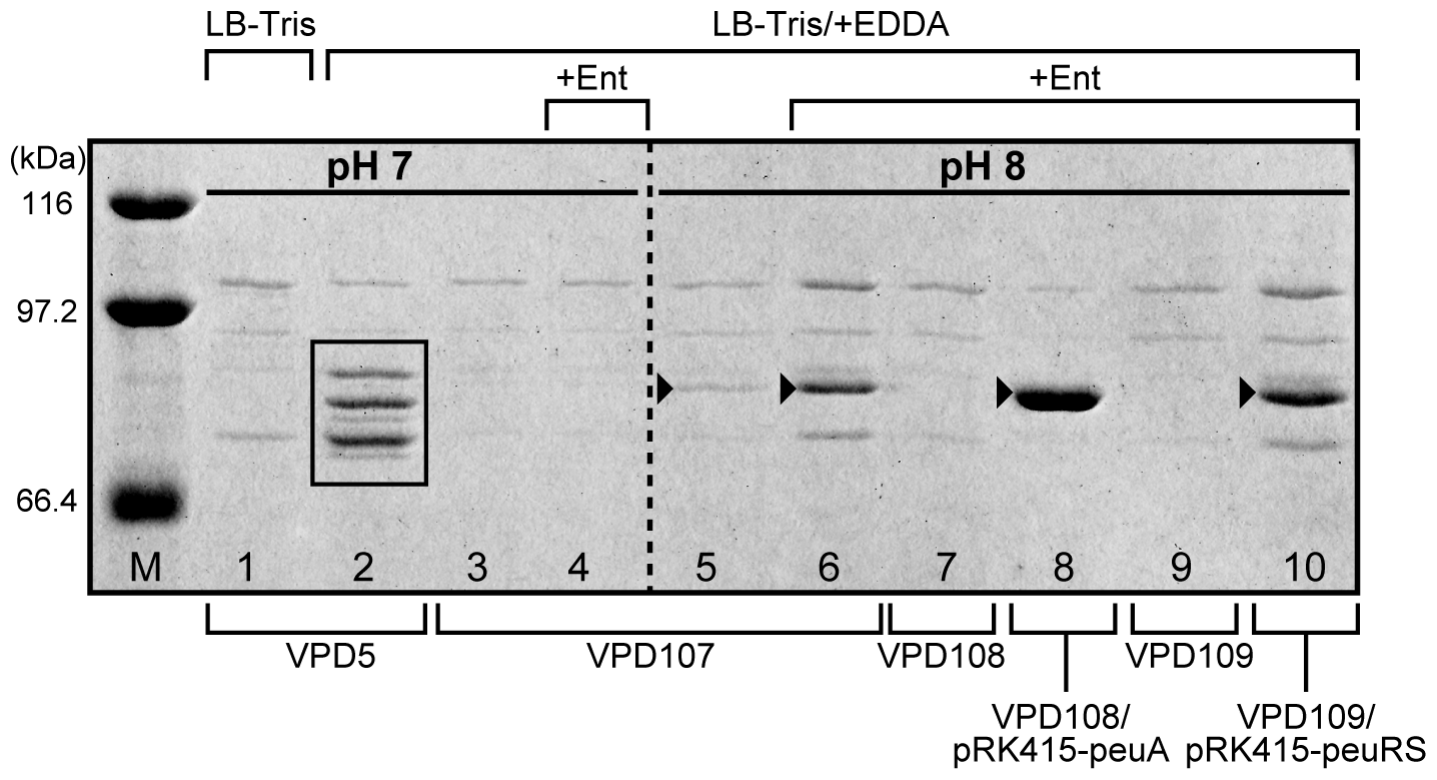
SDS-PAGE analysis of Sarkosyl-insoluble OMPs of *V. parahaemolyticus*. SDS-PAGE analysis was performed with VPD5, VPD107 (seven iron-repressible OMRs-deficient mutant derived from VPD5), VPD108 (*peuA*-deficient mutant derived from VPD107), VPD108/pRK415-peuA, VPD109 (*peuRS*-deficient mutant derived from VPD107), and VPD109/pRK415-peuRS. The OMP fractions were prepared from cells grown in LB-Tris medium at pH 7.0, LB-Tris/+EDDA media at pH 7.0 and 8.0, or LB-Tris/+EDDA/+Ent media at pH 7.0 and 8.0. Lanes 1–7 and 9–10 were loaded with 20 µg OMPs, and lane 8 was loaded with 3 µg OMPs. Electrophoresis was performed on 7.5% SDS-polyacrylamide gels (130 mm long) at a constant current of 15 mA at 4°C. The gel was stained with Coomassie Brilliant Blue. The figure shows only the relevant portions of the gel. The iron-repressible OMPs expressed by VPD5 at pH 7.0 under iron-limiting conditions are boxed in lane 2. Lane M, molecular weight marker proteins; closed arrowheads, PeuA.

**Figure 4:**
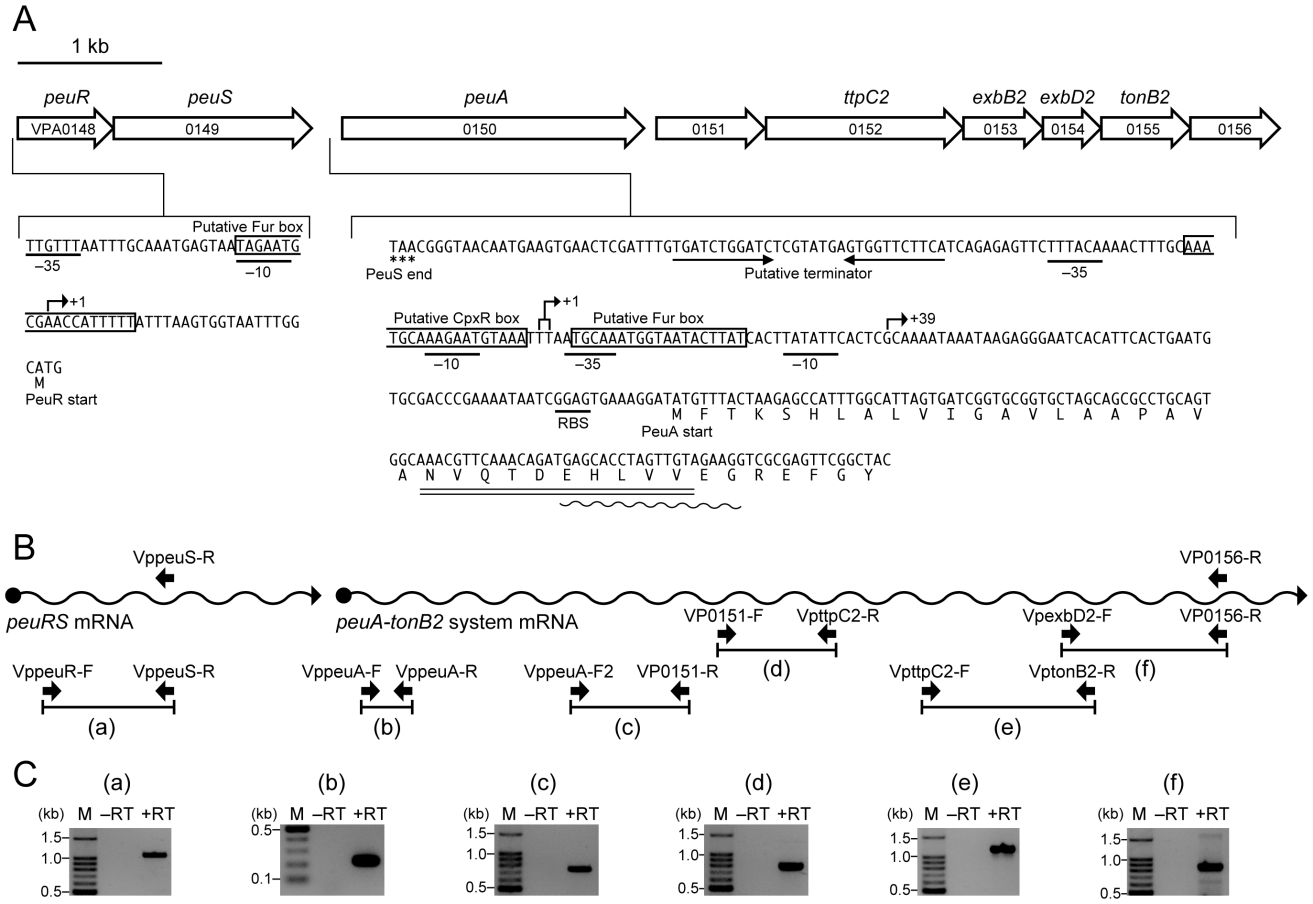
Genetic map and operon structure of *VPA0148*–*VPA0156* locus. (A) Genetic map of the *peuA* gene and the flanking genes. Thick arrows indicate genes and their orientations. The –35 and –10 promoter elements and putative Fur box sequences in the promoter regions of *peuR* (*VPA0148*) and *peuA* (*VPA0150*) are indicated. The transcription start sites for *peuR* (+1) and *peuA* (+1 and +39) are indicated by right-angled arrows. The putative terminator signal between the *peuS* and *peuA* genes, the predicted RBS for the *peuA* gene, the start codons for *peuR* and *peuA* genes, and the stop codon for *peuS* are also indicated. The amino acid sequence consistent with the N-terminal sequence determined for the iron-repressible OMR induced in LB-Tris/+EDDA and LB-Tris/+EDDA/+Ent media at pH 8.0 (see Figure 3) is indicated by a double underline. (B) Schematic representation of mRNAs transcribed from the *VPA0148*-*VPA0156* genes and the primer pairs used for RT-PCR. For preparation of cDNAs by RT, VPpeuS-R and VP0156-R were used. (C) RT-PCR analysis of RT-PCR products. +RT and –RT, RT-PCR was performed with and without reverse transcriptase, respectively. M, 100-bp DNA ladder.

To assess whether extracellular alkaline pH and Ent affect OMP profiles, OMP fractions prepared from *V. parahaemolyticus* mutants were analyzed by SDS-PAGE. The wild-type *V. parahaemolyticus* expresses several iron-repressible OMRs under iron-limiting conditions, as shown in Figure 3, lane 2. We used a VPD107 mutant lacking all of the known iron-repressible OMPs for this experiment, to eliminate potential interference by these OMPs. In LB-Tris/+EDDA medium at pH 7.0, VPD107 was unable to produce PeuA, regardless of Ent presence (Figure 3, lanes 3 and 4). In contrast, when VPD107 was grown in LB-Tris/+EDDA medium at pH 8.0, a faint protein band was detected (Figure 3, lane 5); the sequence of its first 10 N-terminal amino acids was determined to be NVQTDEHLVV. This sequence exactly matched the N-terminal sequence deduced from *peuA* (see Figure 4A), indicating that *peuA* indeed encodes the ferric Ent receptor. The production of PeuA in VPD107 was remarkably increased in LB-Tris/+EDDA+Ent medium at pH 8.0 (Figure 3, lane 6). These results indicate that PeuA is not produced in significant amounts, even under iron-limiting conditions at pH 8.0, unless Ent is present in the growth medium. In addition, VPD108, a *peuA* deletion mutant derived from VPD107, failed to produce PeuA in LB-Tris/+EDDA/+Ent medium at pH 8.0, whereas the complementing strain VPD108/pRK415-peuA restored the ability to produce PeuA (Figure 3, lanes 7 and 8).

### *In silico* Sequence Analyses of *peuA* and Its Adjacent Genes

A map of *peuA* and the neighboring genes is shown in Figure 4A, accompanied by partial nucleotide and deduced amino acid sequences [28]. PeuA shared amino acid similarity with many ferric hydroxamate-type siderophore receptors, such as FhuE (25% identity over 751 amino acids) of *E. coli* [36]; however, it displayed lower sequence similarity with FepA (20% identity over 729 amino acids) and PfeA (23% identity over 226 amino acids), the ferric Ent receptors of *E. coli* [37] and *P. aeruginosa* [13], respectively. A putative Fur box sequence resembling the consensus binding site for the Fur protein in *E. coli* [38] was detected in the promoter region of *peuA* (Figure 4A), and indeed this region cloned on pUC19 was positive in the Fur titration (FURTA) *in vivo* assay [39] (data not shown), indicating that the cloned region harbor the binding site of the *E coli* Fur protein. Moreover, a tandem repeat of 5′-A(N)_3_ GCAAA(N)_4_ GTAAA-3′ (the conserved nucleotides are underlined), termed the CpxR-box [40], [41], which is typical of the CpxR-binding site, was identified in the promoter region of *peuA* (Figure 4A).

Homology searches revealed the existence of putative two-component regulatory system genes (*VPA0148-0149*), collectively named *peuRS*, immediately upstream of *peuA* (Figure 4A). PeuR and PeuS showed amino acid sequence similarity to components of the CpxAR signaling system [42], such as the *P. aeruginosa* PfeR response regulator (36% identity over 225 amino acids) [14] and the *E. coli* CpxA histidine sensor kinase (24% identity over 447 amino acid residues) [43]. The consensus amino acid sequences in the conserved domains of the response and sensor components [13], [15] were also determined for PeuR and PeuS. In addition, they displayed striking structural features, including the presence of invariant amino acid residues, aspartic acid-9 in PeuR and histidine-244 in PeuS, both of which probably serve as phosphorylation sites. The hydropathy profile revealed that PeuS contains two transmembrane-spanning regions and an intervening 115-amino acid extracytoplasmic loop domain that is exposed to the cytoplasmic space. These features of PeuRS are illustrated in Figure S1. Moreover, the cloned promoter region of *peuR* also showed a FURTA-positive phenotype (data not shown), indicative of the presence of the Fur binding site.

### Identification of *peuA*/*VPA0151-0156* and *peuRS* as Iron-Repressible Operons by RT-PCR

The *VPA0151-0156* genes, including the *ttpC2*-*tonB2* system genes, are located downstream of the *peuA* gene, and the open reading frames of the *VPA0151-0156* genes have overlapping stop and start codons, an arrangement typical of transcription unit boundaries in prokaryotic genomes [44]. Although there is a 53-bp gap, including an inverted repeat, immediately downstream of *peuA*, this region contains no potential promoter sequences for the downstream *tonB2* operon (data not shown). As expected, RT-PCR using the primer pairs designed to cover the respective intergenic regions of the *peuA* and *VPA0151*-*0156* genes produced extension bands of the expected size for total RNA prepared from the wild-type strain grown in LB-Tris/+EDDA at pH 7.0 (Figure 4B and C), indicating that these genes are co-transcribed in an iron-regulated operon.

The stop codon of *peuR* overlaps with the start codon of *peuS*, and no definitive promoter sequence was detected upstream of *peuS*. An RT-PCR product of the expected size was also detected for total RNA prepared from the wild-type strain grown in LB-Tris/+EDDA at pH 7.0 when the primer pair VppeuR-F/VppeuS-R was used to amplify the intergenic region between the *peuRS* genes (Figure 4B and C), indicating that these genes also comprise an iron-regulated operon.

### Involvement of the *peuRS* Genes in Ent Utilization at pH 8.0

To investigate the requirement of *peuRS* for Ent utilization at pH 8.0 under iron-limiting conditions, we generated the *peuRS* mutant VPD102 from VPD54, which possesses the *peuA* gene but not the *vctA* and *irgA* genes, for use in a growth assay. While VPD102 abolished the Ent-mediated growth observed for VPD54 in LB-Tris/+EDDA medium at pH 8.0, the complementing strain VPD102/pRK415-peuRS grew well in the same medium (Figure 2). Furthermore, to examine the effect of *peuRS* on PeuA production by SDS-PAGE, the VPD109 mutant with the *peuRS* operon deleted was constructed from VPD107. VPD109 showed a complete lack of PeuA, even in LB-Tris/+EDDA/+Ent medium at pH 8.0 (Figure 3, lane 9); however, the complementing strain VPD109/pRK415-peuRS restored the ability to produce PeuA in the same medium (Figure 3, lane 10). Taken together, these data highlight a possible role of the PeuRS two-component regulatory system in the production of PeuA induced by alkaline pH and Ent.

### Iron-Repressible Transcription of *peuR*

**Figure 5:**
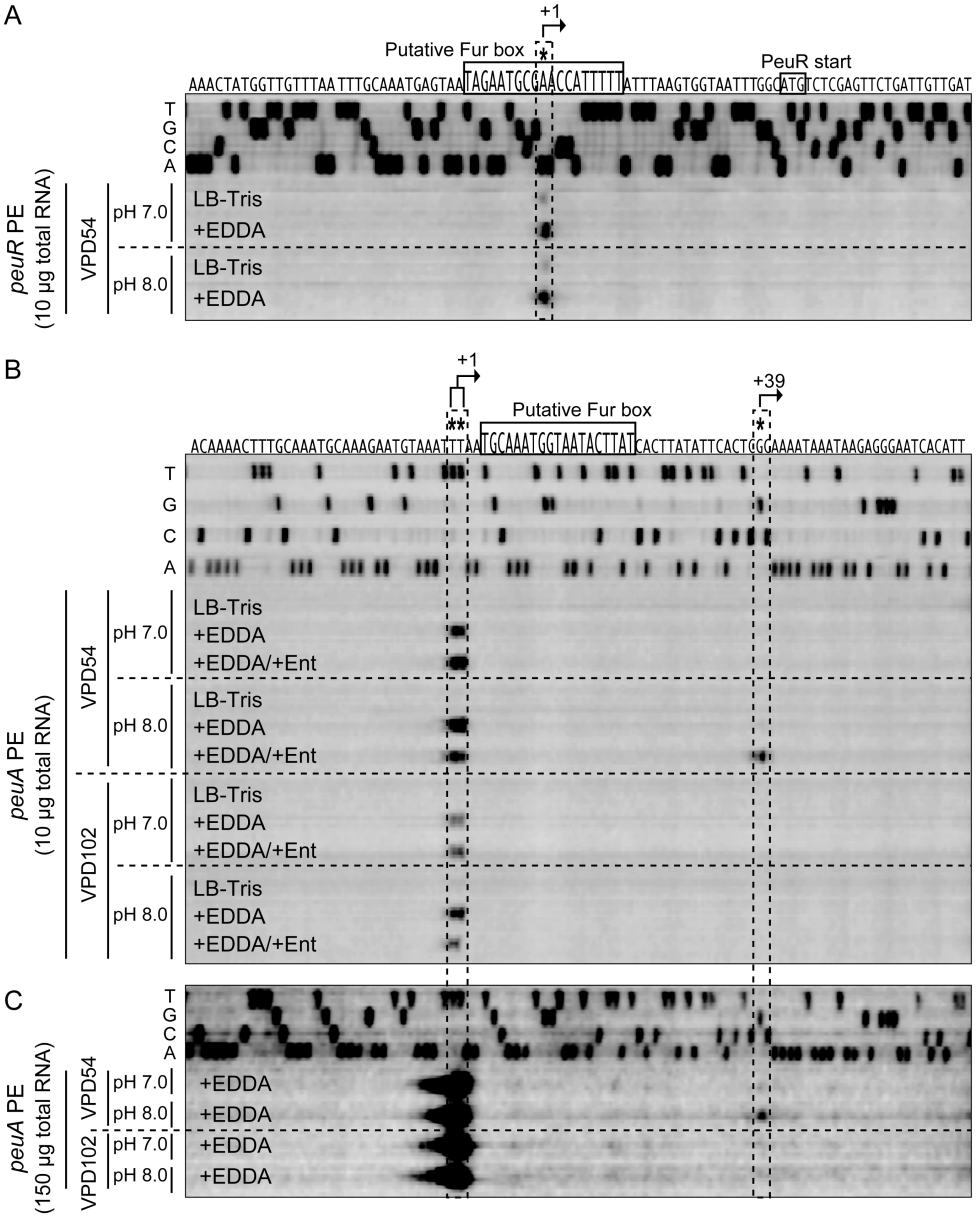
Primer extension analyses of total RNA from VPD54 or VPD102 to determine the transcription start sites of *peuR* (A) and *peuA* (B and C). Total RNAs were isolated from VPD54 (*vctA*- and *irgA*-deficient mutant derived from VPD5) and VPD102 (*peuRS*-deficient mutant derived from VPD54) grown at pH 7.0 and 8.0 in LB-Tris, LB-Tris/+EDDA, and LB-Tris/+EDDA/+Ent media. The amounts of total RNA and primers used for reverse transcription were as follows: (A) 10 µg VppeuR-PE, (B) 10 µg VppeuA-PE, and (C) 150 µg VppeuA-PE. The same primers used for primer extension analysis were used to generate the sequence ladders (A, C, G, T). The transcription start sites and putative Fur boxes are indicated at the top of panels A and B (also see Figure 3A).

To determine the transcriptional start site for *peuR*, and to test whether its expression is iron-regulated, primer extension analysis was also performed for total RNA samples of VPD54 cells grown in LB-Tris and LB-Tris/+EDDA media. The transcription of *peuR* was unambiguously derepressed in LB-Tris/+EDDA medium, independent of pH (Figure 5A), and the transcription start site (+1) of *peuR* was determined to be 29 nucleotides upstream of its start codon (Figure 4A). It is evident from these data that the *peuRS* operon is constitutively expressed under iron-limiting conditions.

### Induction of an Alternative Transcript of *peuA* under Iron-Limiting Conditions in Response to Extracellular Alkaline pH and Ent

Primer extension analyses were performed with total RNA samples of both VPD54 and its *peuRS* deletion mutant VPD102. Two primer extension products were detected, when total RNA samples prepared from VPD54 grown in LB-Tris/+EDDA media at pH 7.0 and 8.0 or LB-Tris/+EDDA/+Ent medium at pH 7.0 were used as the templates. However, the growth of VPD54 was not promoted under the same conditions. Two probable transcription start sites of *peuA* were mapped to 105 bp and 104 bp upstream of the *peuA* ATG start codon (Figure 5B); hereafter, the transcripts from the +1 and +2 sites are collectively referred to as the +1 transcript. Another transcription start site for *peuA* (+39 transcript) was detected 67 bp upstream of its start codon, when VPD54 was grown in LB-Tris/+EDDA/+Ent medium at pH 8.0 (Figure 5B). In addition, the growth of VPD54 was promoted under the same conditions. These results suggested that the +1 transcript is not responsible for the growth of VPD5 as shown in Figure 1. Interestingly, the primer extension product of the +39 transcript was not detected when VPD102 was grown in the same medium at pH 7.0 (Figure 5B). However, when primer extension analysis was performed using a 15-fold excess of RNA, a small amount of the primer extension product of the +39 transcript was also detected in VPD54 cells grown in LB-Tris/+EDDA medium at pH 8.0, even when Ent was absent from the medium (Figure 5C). This is consistent with the expression of PeuA in VPD107 grown under the same conditions (Figure 3, lane 5), and this low level of PeuA expression in VPD107 under iron-limiting conditions may contribute to the initial uptake of ferric Ent for stimulation of PeuS prior to induction of *peuA* expression at pH 8.0. However, no extension band was detected for total RNA prepared from VPD54 grown in LB-Tris medium, suggesting that the putative Fur box detected in the promoter region of *peuA* (Figure 4A) was functional for the iron-repressive regulation of *peuA*. Collectively, these findings indicate that *peuA* mRNA is transcribed as the +1 transcript under iron-limiting conditions irrespective of Ent and pH, while the +39 transcript responsible for the expression of PeuA is expressed in trace amounts in LB-Tris/+EDDA medium at pH 8.0 and is significantly increased by the presence of Ent under the same conditions. These data also suggested that the increase in the +39 transcript was absolutely dependent on the PeuRS two-component regulatory system. In addition, two sets of the −35 and −10 promoter sequences are properly positioned for transcription from the +1 and +39 sites (Figure 4A).

**Figure 6:**
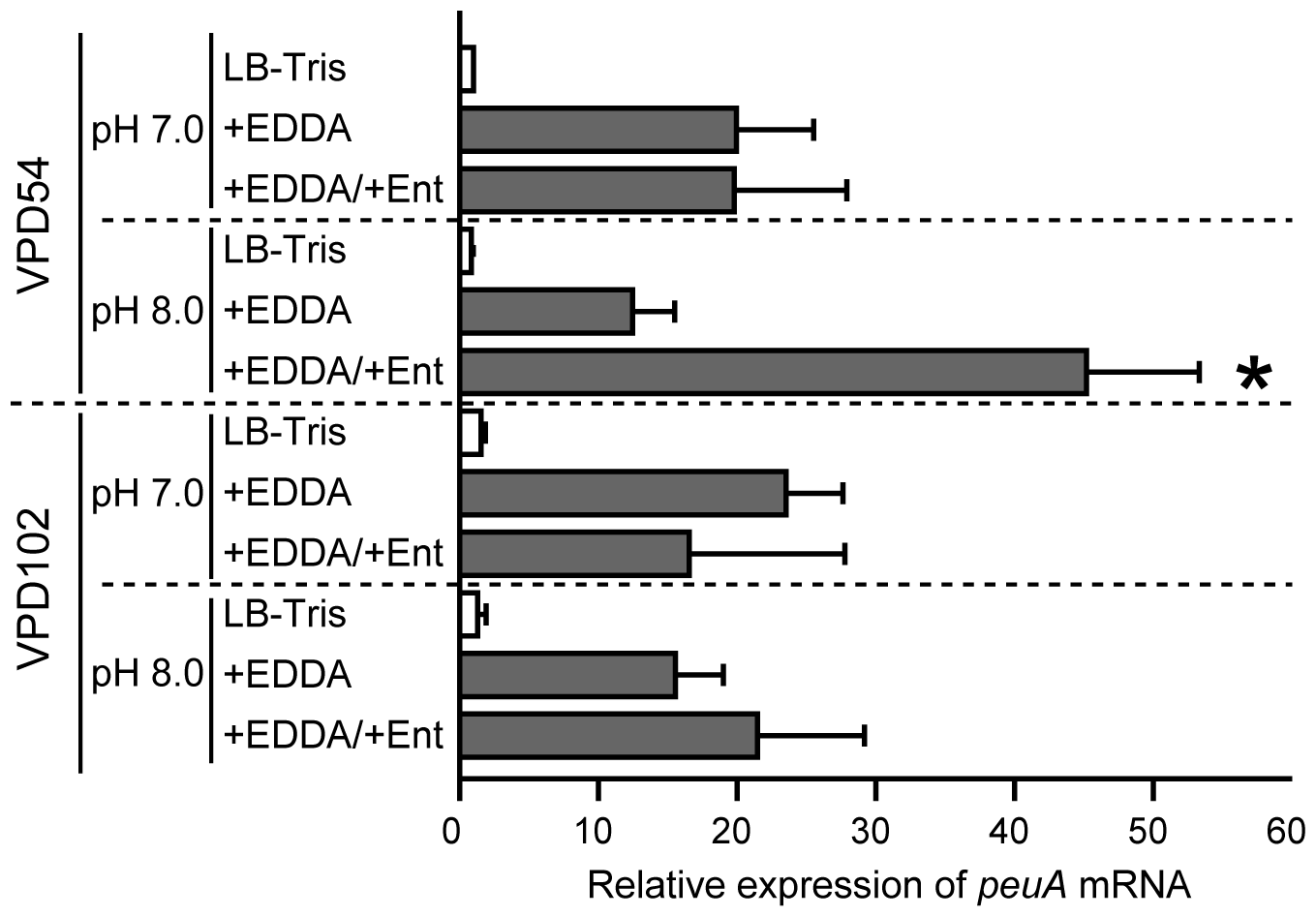
Relative levels of *peuA* mRNA, assessed by RT-qPCR. Total RNA samples were prepared from VPD54 (*vctA*- and *irgA*-deficient mutant derived from VPD5) and VPD102 (*peuRS*-deficient mutant derived from VPD54) grown at pH 7.0 and 8.0 in LB-Tris, LB-Tris/+EDDA, and LB-Tris/+EDDA/+Ent media. Data are shown as means ± SD from 3 separate experiments. An asterisk indicates *P*<0.05 compared to other samples.

To confirm the iron-regulated expression of *peuA*, RT-qPCR analysis was performed. At pH 7.0 and 8.0, the transcription of *peuA* was strongly induced in VPD54 and VPD102 in LB-Tris/+EDDA medium (10- to 20-fold increases compared to the level in LB-Tris medium) (Figure 6). Moreover, in LB-Tris/+EDDA medium at pH 7.0, VPD54 produced *peuA* mRNA at similar levels in the presence and absence of Ent; however, the addition of Ent to LB-Tris/+EDDA medium at pH 8.0 conspicuously increased the level of *peuA* mRNA (Figure 6). Considering the results of primer extension analysis (Figure 5B), these data suggested that the increase in the *peuA* mRNA was due to transcription from the +39 site. No such effect of Ent on *peuA* transcription at pH 8.0 was observed for the *peuRS*-deletion mutant VPD102 (Figure 6), implying that the *peuRS* operon is responsible for the transcription of *peuA* from the +39 site.

### mRNA Secondary Structure Prediction

**Figure 7:**
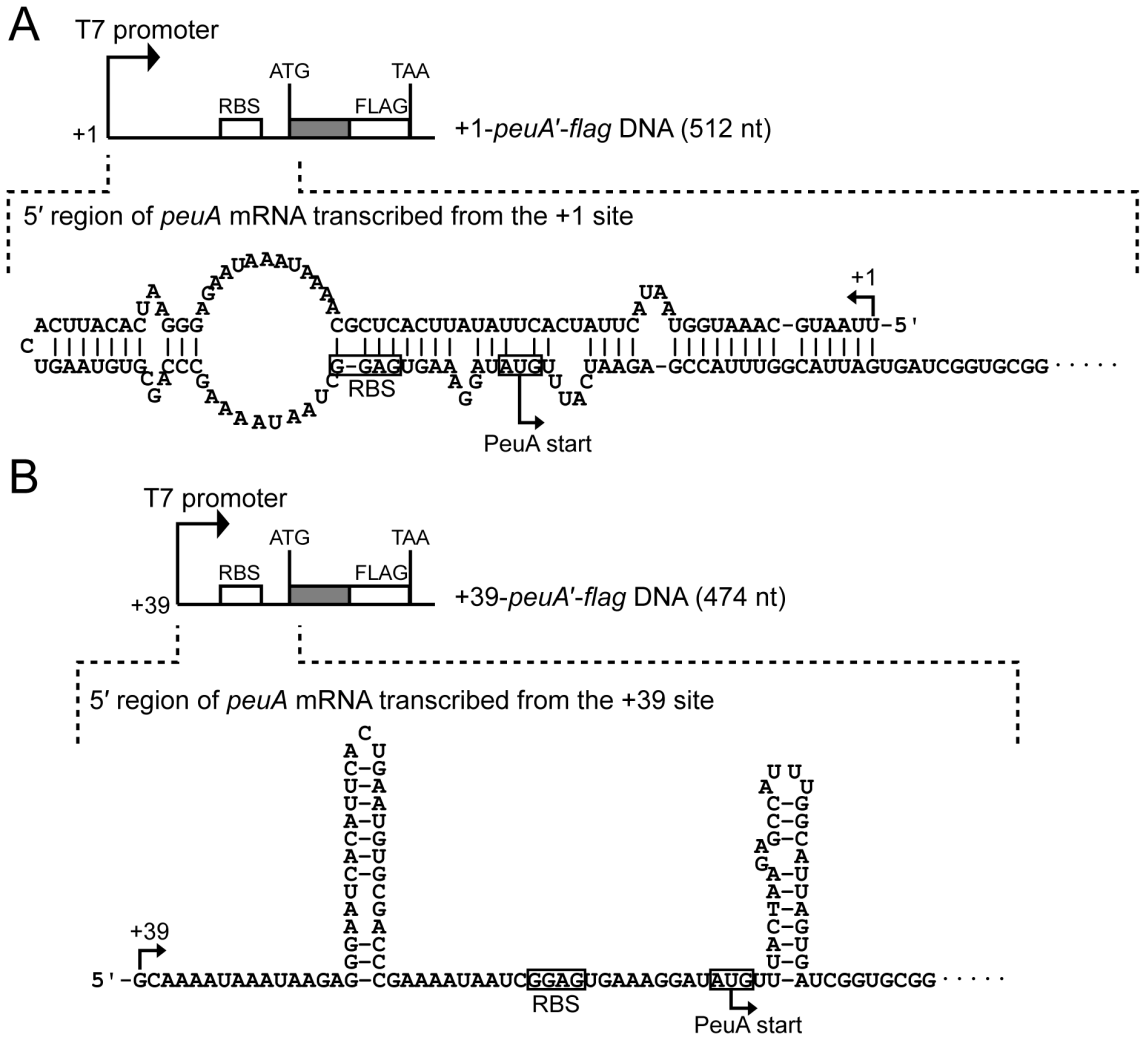
Schematic representation of the +1-*peuA*′-*flag* (A) and +39-*peuA*′-*flag* (B) DNA fragments. Each of these DNA fragments includes a nucleotide sequence corresponding to the *peuA* 5′-UTR from the +1 or +39 sites and the nucleotide sequence for the N-terminal 99 amino acid residues (in gray), in addition to a T7 promoter and a FLAG tag preceding the stop codon (TAA). The secondary structures of the 5′-UTRs of the +1 transcript (A) and the +39 transcript (B) of *peuA* are shown, both of which were predicted by the CentroidFold software (http://www.ncrna.org/centroidfold/). The RBS and start codon of *peuA* mRNA are boxed in the secondary structures.

The results shown in Figure 3 indicate that remarkable amounts of PeuA are produced under iron-limiting conditions in response to an extracellular pH of 8.0, but not 7.0, and Ent. This corresponds with appearance of the +39 transcript, in addition to +1 transcription, under the same growth conditions. These findings led us to hypothesize that these transcripts might contain regulatory signals in their 5′-untranslated regions (UTRs) that couple transcription to translation. Thus, to better define the nature of these transcripts, the secondary structures of their 5′-UTRs were predicted using the CENTROIDFOLD program (http://www.ncrna.org/centroidfold/) [45]. Figure 7A shows that the first 40 nucleotides of the 5′-UTR of the *+*1 transcript are folded into the secondary structure with the ribosomal binding site (RBS) and the start codon of *peuA* to block initiation of translation. As opposed to the +1 transcript, the +39 transcript does not form an inhibitory structure in its 5′-UTR, thus allowing initiation of translation (Figure 7B). Therefore, we hypothesized that PeuA production might be dependent on the translation of the +39 *peuA* transcript.

### The +39 Transcript is Responsible for PeuA Production

**Figure 8:**
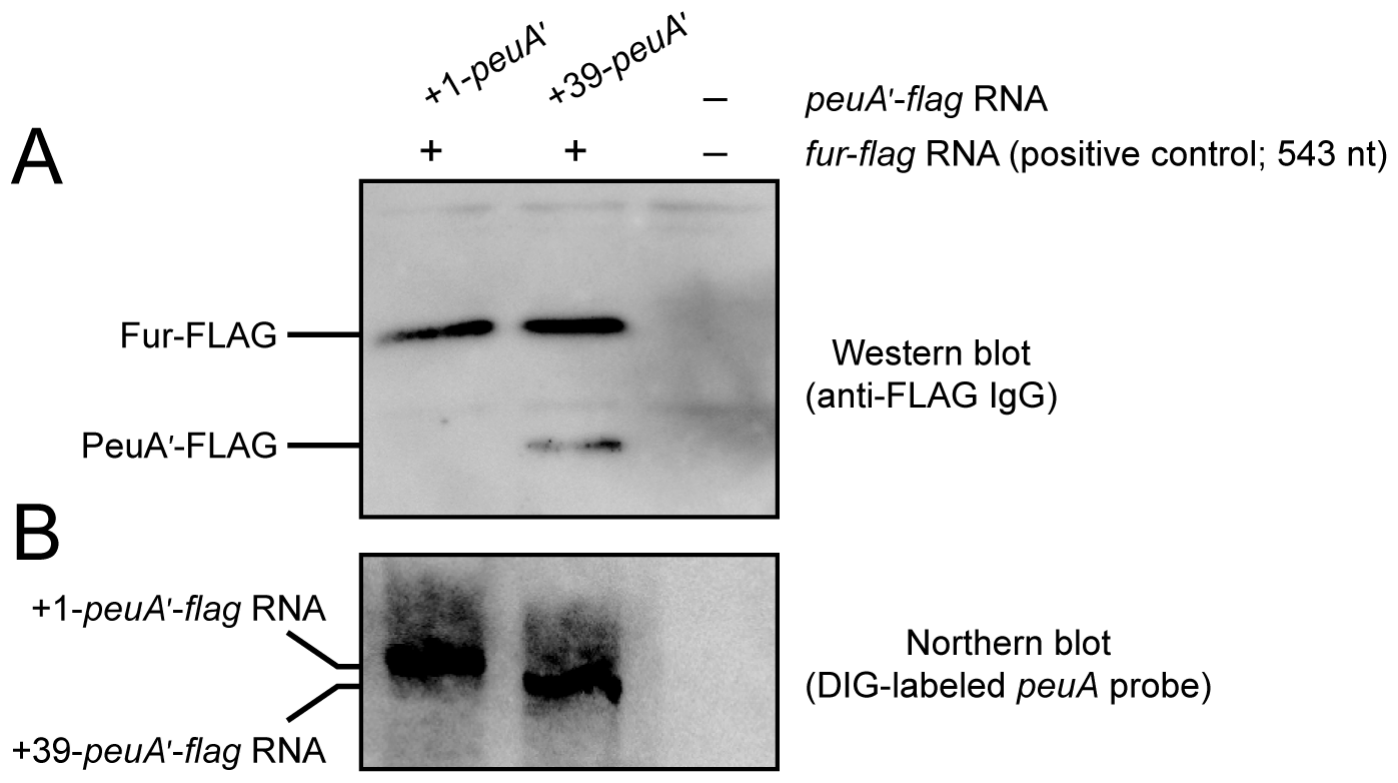
*In vitro* translation of *peuA* mRNA. (A) *In vitro* translation analysis of the +1 and +39 *peuA* transcripts labeled with the FLAG tag. The +1-*peuA*′-*flag* RNA and +39-*peuA*′-*flag* RNA were first synthesized by *in vitro* transcription, as described in the MATERIALS AND METHODS, and a mixture containing either the +1-*peuA*′-*flag* RNA (30 pmol)/*fur*-*flag* RNA (3 pmol) or the +39-*peuA*′-*flag* RNA (30 pmol)/*fur*-*flag* RNA (3 pmol) as the template was subjected to *in vitro* translation. The FLAG-fused proteins translated were separated on 15% SDS-polyacrylamide gels, and were detected by western blotting using anti-FLAG IgG. (B) Confirmation of the presence of +1-*peuA*′-*flag* RNA and +39-*peuA*′-*flag* RNA in the reaction mixture for *in vitro* translation. These RNA fragments were detected in the reaction mixture by northern blotting using a DIG-labeled *peuA* probe.

To test the above hypothesis, an *in vitro* translation assay was performed. The RNA templates for *in vitro* translation, i.e., +1-*peuA*′-*flag* (Figure 7A), +39-*peuA*′-*flag* (Figure 7B), and *fur*-*flag* RNAs, were constructed by *in vitro* transcription using DNA templates containing the T7 promoter. The *in vitro* translation products were analyzed by SDS-PAGE followed by western blotting using anti-FLAG IgG. The PeuA′-FLAG product was not detected when a mixture of +1-*peuA*′-*flag* RNA and *fur-flag* RNA was used for *in vitro* translation, even though Fur-FLAG, a positive control for *in vitro* translation, was detected; however, when a mixture of +39-*peuA*′*-flag* RNA and *fur-flag* RNA was used as the template, a significant amount of PeuA′-FLAG was detected along with Fur-FLAG (Figure 8A). Simultaneously, the +1-*peuA*′-*flag* RNA and +39-*peuA*′*-flag* RNA in each reaction mixture for *in vitro* translation were validated by northern blotting using a DIG-labeled *peuA* probe (Figure 8B). These data are concordant with the secondary structures predicted for the +1 and +39 transcripts of *peuA*.

### TonB Specificity of PeuA

*V. parahaemolyticus* contains up to three TonB clusters encoding TonB1 and TonB2 systems on the small chromosome and a TonB3 system on the large chromosome, and the TonB2 system is located downstream of *peuA*. To determine which TonB systems are involved in the transport of ferric Ent via PeuA, a set of isogenic *tonB* deletion mutants were constructed from VPD54. A growth assay showed that only the VPD73 mutant deficient in *tonB2* lost the ability to grow in LB-Tris/+EDDA/+Ent medium at pH 8.0; however, the VPD72 and VPD74 mutants deficient in *tonB1* and *tonB3*, respectively, grew as well as their parental strain, VPD54, in the same medium (Figure S2). These observations show that the TonB2 system functions as an energy modulator for PeuA.

### Distribution of Orthologs of the *V. parahaemolyticus PeuRSA-VPA0151-VPA0156* Genes Among Other *Vibrio* Species

**Figure 9:**
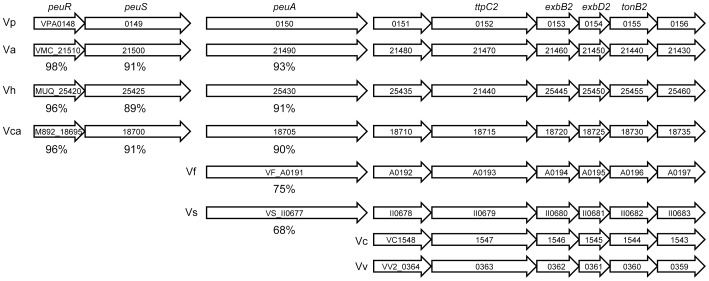
Distribution of the *Vibrio parahaemolyticus VPA0148*–*VPA0156* orthologs in other *Vibrio* species for which whole-genomic sequences have been reported. Arrows represent genes and their orientations. The numbers below the genes indicate percent amino acid sequence similarities to *V. parahaemolyticus* PeuRS and PeuA. Vp, *V. parahaemolyticus*; Va, *V. alginolyticus*; Vh, *V. harveyi*; Vca, *V. campbellii*; Vf, *V. fischeri*; Vs, *V. splendidus*; Vc, *V. cholerae*; Vv, *V. vulnificus*.

Using BLAST analyses, we examined whether orthologs to the *V. parahaemolyticus peuRSA-VPA0151–VPA0156* locus genes are distributed among the whole-genome sequences of other *Vibrio* species. Although the *VPA0151–VPA0156* (the *ttpC2*-*tonB2* system genes) cluster was identified in all *Vibrio* species examined, *peuRS* and *peuA* orthologs were identified only in *V. alginolyticus, V. harveryi*, and *V. campbellii*, which belong to the same phylogenetic group (the *V. harveyi* group) as *V. parahaemolyticus* [20] (Figure 9). However, all *peu* orthologs are absent from *V. chorelae* and *V. vulnificus*, and *V. fischeri* and *V. splendidus* possess the *peuA* ortholog, but not the *peuRS* orthologs.

## Discussion

Based on the results obtained in this study, we propose a mechanism for enhanced expression of the *V. parahaemolyticus peuA* through the action of PeuRS in response to extracellular alkaline pH and Ent (Figure S3). Our data showed that *peuA* is transcribed in a polycistronic mRNA with the *VPA0151*–*VPA0156* genes under iron-limiting conditions (Figure 4B). However, at neutral pH, translation of the +1 *peuA* transcript appeared to be inhibited by the formation of a secondary structure in its 5′-UTR that blocks the RBS and start codon of *peuA* (Figure 7A); in contrast, the *VPA0151-VPA0156* mRNA responsible for the TonB2 system was expected to be translated normally under the same conditions, because the RBSs and start codons of their genes are available for ribosomal access. This expectation is supported by the fact that the *V. parahaemolyticus* PvuA1 is exclusively dependent on the energy transduced by the TonB2 system [24] and remains fully functional as the ferric vibrioferrin receptor under iron-limiting conditions at neutral pH. When grown under iron-limiting conditions at pH 8.0, *V. parahaemolyticus* expressed the *peuA*-*VPA0151*–*VPA0156* polycistronic mRNA from the +39 start site that was induced via the two-component regulatory system, PeuRS (Figure 5B). No inhibitory secondary structure was identified in the 5′-UTR of the +39 transcript; in other words, PeuA is synthesized owing to the translation of the +39 transcript (Figure 7B). Moreover, as shown in Figures 3, 5, and 6, the levels of PeuA and the +39 transcript expressed under iron-limiting conditions were more markedly elevated in the presence than in the absence of Ent. These observations indicate that Ent functions as a potent inducer for transcription from the +39 site, with the aid of PeuRS.

A number of Gram-negative bacteria, including *P. aeruginosa* [46], *Bordetella* spp. [47], *V. cholerae* [48], *V. anguillarum* [49], *V*. *parahaemolyticus* [27], and *Neisseria gonorrhoease* [50], are known to utilize Ent as a xenosiderophore, which induces the cognate ferric Ent receptors under iron-limiting conditions. Such a wide distribution of this system in bacteria may be explained by the fact that Ent has an exceptionally high affinity for ferric iron, and its production by bacterial species is wider than previously thought [51]; Ent has been reported to be synthesized and excreted by most enterics [52], as well as two Gram-positive *Streptomyces* species [53]. Moreover, ferric Ent is more stable at alkaline pH than ferric hydroxamate-type siderophores [54]. In the pathogens described above, except for *P. aeruginosa*, the AraC-like or LysR-like transcriptional regulators operate to induce these ferric Ent receptors. *P. aeruginosa* has been reported to utilize Ent through the two-component regulatory system PfeRS [13], [14]; however, expression of the ferric Ent receptor (PfeA) in this species is enhanced in response to Ent under iron-limiting conditions [46], and only a single set of promoter sequences (−10 and −35) are present in the region upstream of *pfeA* [14]. Another unique two-component regulatory system, operating through heme-dependent regulation, has been described in the Gram-positive bacterium *Corynebacterium diphtheriae* for the expression of a heme oxygenase gene responsible for the utilization of heme as an iron source [55]. In these systems, the signal molecules likely interact with the sensors, leading to activation of the response regulators.

The *E. coli* CpxAR two-component regulatory system is well known to be involved in counteracting extracellular stresses, including alkaline pH exposure [56], [57]. Extracellular signals cause a conformational change in CpxA, stimulating the autophosphorylation of a conserved histidine residue. Once this residue is phosphorylated, CpxA acts as a kinase and phosphorylates a conserved aspartate residue on CpxR. Phosphorylated CpxR acts on its target gene as a transcriptional activator [42]. Considering that PeuRS is homologous to members of the Cpx signaling system, it seems likely that the conformation of PeuS is altered to initiate the signal transduction cascade in response to an extracellular alkaline pH and Ent, although it is not known whether these stimuli interact with PeuS separately or cooperatively. The activated PeuS phosphorylates PeuR, and the resulting phosphorylated PeuR is expected to bind to the *peuA* promoter region to induce transcription from the +39 site. However, it remains unclear whether additional factor(s) are required to transduce the signals of extracellular alkaline pH and Ent.

*V. parahaemolyticus* has also been reported to utilize Ent through two other ferric Ent receptors, VctA and IrgA [27]. In this study, these receptors were ascertained to operate under iron-limiting conditions at pH 8.0 (see Figure S4). Therefore, it is likely that the PeuA-mediated Ent utilization system is substituted and/or supplemented by Ent utilization via VctA and IrgA, and vice versa, signifying that, in bacteria, the expression of multiple siderophore receptors may be a common strategy or a backup system to capture the iron essential for survival and proliferation. Moreover, from evolutionary and ecological points of view, it is of interest that the *peuRSA* cluster is restrictively distributed in the phylogenetic group that includes *V. alginolyticus*, *V. harveyi*, and *V. campbellii* in addition to *V. parahaemolyticus*, all of which live in marine or estuarine environments at a pH of approximately 8.1 [58], often in association with plankton or animals, including fish and shellfish [19], [20]. However, it is uncertain whether *V. parahaemolyticus* and the other species naturally encounter Ent. Alternatively, the authentic ligand for PeuA and PeuS could be another siderophore structurally similar to Ent that is produced by microorganisms inhabiting the same niches as *V. parahaemolyticus*.

In conclusion, our study establishes that under iron-limiting conditions, the *V. parahaemolyticus* two-component regulatory system PeuRS functions in concert with extracellular alkaline pH and Ent for the induction of *peuA* transcription at the +39 site, leading to production of PeuA. Further studies are needed to clarify the molecular mechanisms by which the PeuRS two-component system is activated in response to extracellular alkaline pH and Ent to induce transcription beginning at the +39 site.

## Supporting Information

Figure S1 **Amino acid sequences of PeuR (A) and PeuS (B).** Consensus amino acid residues in the conserved regions are boxed, and invariant amino acid residues (proposed to be important for the function of PeuRS) are indicated by asterisks. In panel B, transmembrane (TM) helices proposed by the HMMTOP transmembrane topology prediction server (http://www.enzim.hu/hmmtop/index.php) are underlined. (C) A hydropathy plot of PeuS. The hydropathic index was calculated by the method of Kyte and Doolittle using a window of 21 amino acid residues. Solid bars correspond to the TM helices shown in panel B.
(PDF)

Figure S2 **TonB specificity of PeuA in Ent utilization.** The growth assay was performed as described in Figure 1. Data are shown as means ± SD from 3 separate experiments.
(PDF)

Figure S3 **Proposed expression mechanism for** ***V. parahaemolyticus*** **PeuA ferric Ent receptor under iron-limiting conditions in response to extracellular alkaline pH and Ent.** Thick arrows and wavy arrows represent the open reading frames and the direction of transcription and mRNAs, respectively. (A) Under iron-limiting conditions at pH 7.0, *peuA* is co-transcribed with *VPA0151*-*VPA0156* from the +1 transcription start site. However, the transcript from the +1 site forms a secondary structure within its 5′-UTR, leading to inhibition of translation of the *peuA* mRNA, although the remaining *VPA0151*–*VPA0156* mRNA is translated. (B) Under iron-limiting conditions at pH 8.0 in the absence of Ent, transcription of the *peuA*/*VPA0151*–*VPA0156* operon from the +39 site also occurs to a slight extent, combined with normal transcription beginning at the +1 site. The presence of Ent under iron-limiting conditions at pH 8.0 is proposed to result in induction of transcription from the +39 site, and thereby leads to enhanced expression of the ferric Ent receptor PeuA, because the RBS and start codon of *peuA* in the +39 transcript are available for translation initiation. The *peuA* gene, therefore, is optimally expressed under iron-limiting conditions in response to extracellular alkaline pH and Ent. In addition, the two-component regulatory system, PeuRS, is proposed to be necessary to activate *peuA* transcription in response to these signals.
(PDF)

Figure S4 **Growth assays of the VPD54, VPD55, VPD56, and VPD57 mutants in LB-Tris/+EDDA/+Ent medium at pH 8.0.** The growth assay was performed as described in Figure 1. Data are shown as means ± SD from 3 separate experiments.
(PDF)

Table S1 **Plasmids used in this study.**
(PDF)

Table S2 **PCR primers used in this study.**
(PDF)
