# Supplementary material for: Regulation of the Expression of the Vibrio parahaemolyticus peuA Gene Encoding an Alternative Ferric Enterobactin Receptor
Source: PLoS One. 2014 Aug 22;9(8):e105749. doi: 10.1371/journal.pone.0105749 (PMC4141801; doi:10.1371/journal.pone.0105749)
Supplement: Figure S1 — Amino acid sequences of PeuR (A) and PeuS (B). Consensus amino acid residues in the conserved regions are boxed, and invariant amino acid residues (proposed to be important for the function of PeuRS) are indicated by asterisks. In panel B, transmembrane (TM) helices proposed by the HMMTOP transmembrane topology prediction server (http://www.enzim.hu/hmmtop/index.php) are underlined. (C) A hydropathy plot of PeuS. The hydropathic index was calculated by the method of Kyte and Doolittle using a window of 21 amino acid residues. Solid bars correspond to the TM helices shown in panel B. (PDF) [file pone.0105749.s001.pdf]

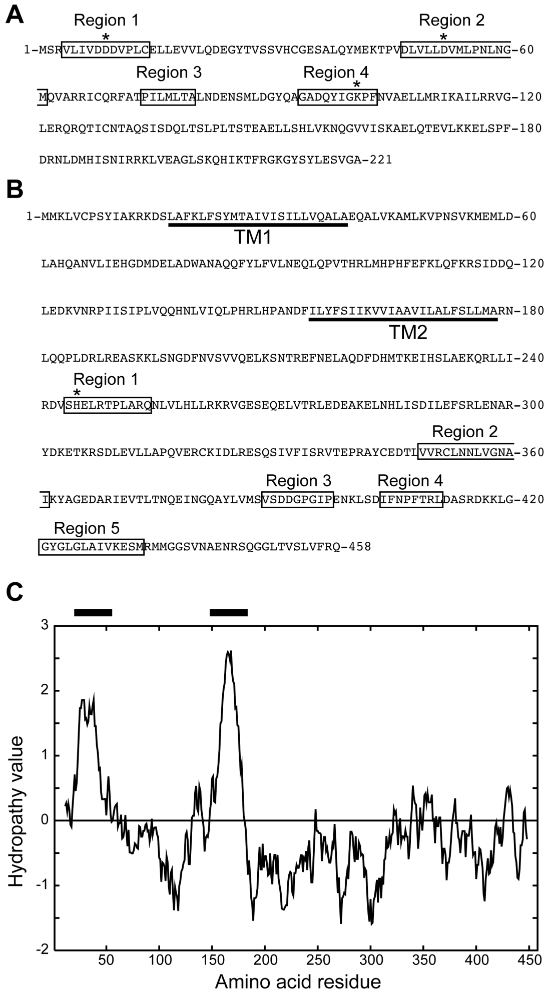

Figure S1. Amino acid sequences of PeuR (A) and PeuS (B). Consensus amino acid residues in the conserved regions are boxed, and invariant amino acid residues (proposed to be important for the function of PeuRS) are indicated by asterisks. In panel B, transmembrane (TM) helices proposed by the HMMTOP transmembrane topology prediction server (http://www.enzim.hu/hmmtop/index.php) are underlined. (C) A hydropathy plot of PeuS. The hydropathic index was calculated by the method of Kyte and Doolittle using a window of 21 amino acid residues. Solid bars correspond to the TM helices shown in panel B.
