# Supplementary material for: Regulation of the Expression of the Vibrio parahaemolyticus peuA Gene Encoding an Alternative Ferric Enterobactin Receptor
Source: PLoS One. 2014 Aug 22;9(8):e105749. doi: 10.1371/journal.pone.0105749 (PMC4141801; doi:10.1371/journal.pone.0105749)
Supplement: Figure S3 — Proposed expression mechanism for V. parahaemolyticus PeuA ferric Ent receptor under iron-limiting conditions in response to extracellular alkaline pH and Ent. Thick arrows and wavy arrows represent the open reading frames and the direction of transcription and mRNAs, respectively. (A) Under iron-limiting conditions at pH 7.0, peuA is co-transcribed with VPA0151-VPA0156 from the +1 transcription start site. However, the transcript from the +1 site forms a secondary structure within its 5′-UTR, leading to inhibition of translation of the peuA mRNA, although the remaining VPA0151–VPA0156 mRNA is translated. (B) Under iron-limiting conditions at pH 8.0 in the absence of Ent, transcription of the peuA/VPA0151–VPA0156 operon from the +39 site also occurs to a slight extent, combined with normal transcription beginning at the +1 site. The presence of Ent under iron-limiting conditions at pH 8.0 is proposed to result in induction of transcription from the +39 site, and thereby leads to enhanced expression of the ferric Ent receptor PeuA, because the RBS and start codon of peuA in the +39 transcript are available for translation initiation. The peuA gene, therefore, is optimally expressed under iron-limiting conditions in response to extracellular alkaline pH and Ent. In addition, the two-component regulatory system, PeuRS, is proposed to be necessary to activate peuA transcription in response to these signals. (PDF) [file pone.0105749.s003.pdf]

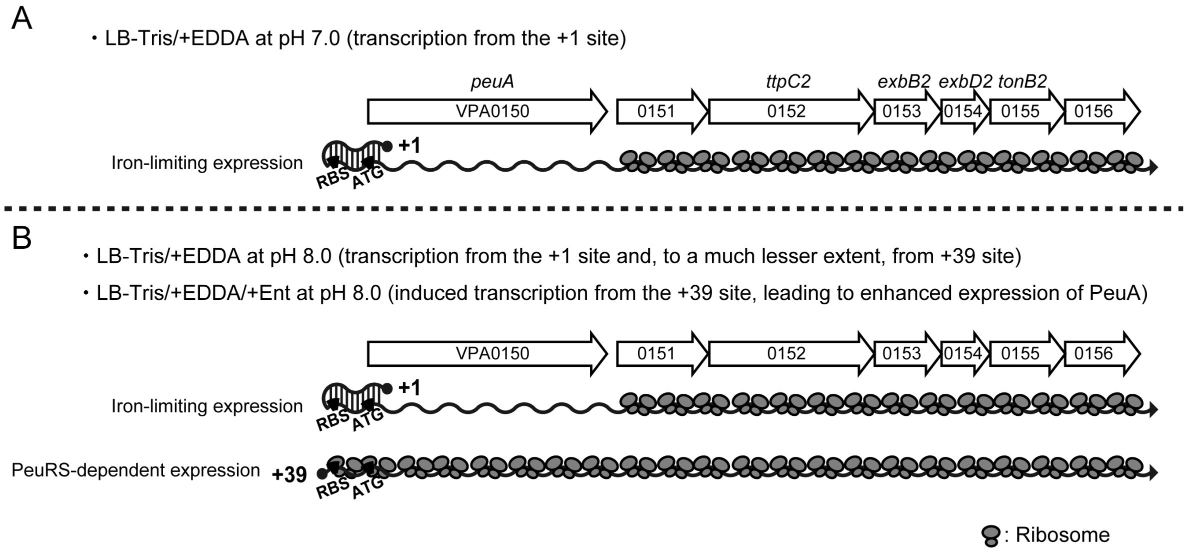

Figure S3. Proposed expression mechanism for *V. parahaemolyticus* PeuA ferric Ent receptor under iron-limiting conditions in response to extracellular alkaline pH and Ent. Thick arrows and wavy arrows represent the open reading frames and the direction of transcription and mRNAs, respectively. (A) Under iron-limiting conditions at pH 7.0, *peuA* is co-transcribed with *VPA0151-VPA0156* from the +1 transcription start site. However, the transcript from the +1 site forms a secondary structure within its 5′-UTR, leading to inhibition of translation of the *peuA* mRNA, although the remaining *VPA0151–VPA0156* mRNA is translated. (B) Under iron-limiting conditions at pH 8.0 in the absence of Ent, transcription of the *peuA*/*VPA0151–VPA0156* operon from the +39 site also occurs to a slight extent, combined with normal transcription beginning at the +1 site. The presence of Ent under iron-limiting conditions at pH 8.0 is proposed to result in induction of transcription from the +39 site, and thereby leads to enhanced expression of the ferric Ent receptor PeuA, because the RBS and start codon of *peuA* in the +39 transcript are available for translation initiation. The *peuA* gene, therefore, is optimally expressed under iron-limiting conditions in response to extracellular alkaline pH and Ent. In addition, the two-component regulatory system, PeuRS, is proposed to be necessary to activate *peuA* transcription in response to these signals.
