# Supplementary material for: Regulation of the Expression of the Vibrio parahaemolyticus peuA Gene Encoding an Alternative Ferric Enterobactin Receptor
Source: PLoS One. 2014 Aug 22;9(8):e105749. doi: 10.1371/journal.pone.0105749 (PMC4141801; doi:10.1371/journal.pone.0105749)
Supplement: Figure S4 — Growth assays of the VPD54, VPD55, VPD56, and VPD57 mutants in LB-Tris/+EDDA/+Ent medium at pH 8.0. The growth assay was performed as described in Figure 1. Data are shown as means ± SD from 3 separate experiments. (PDF) [file pone.0105749.s004.pdf]

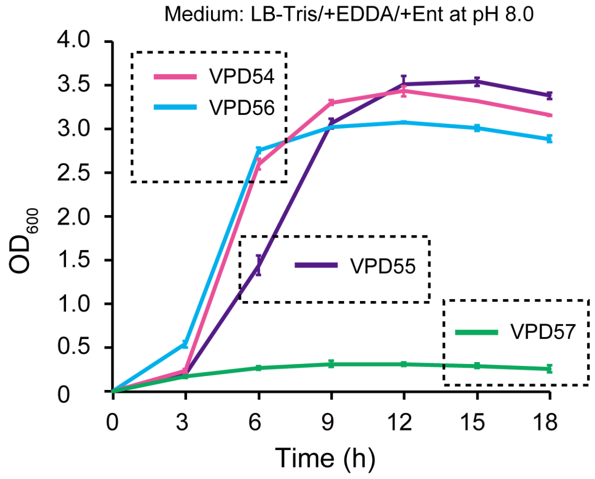

Figure S4. Growth assays of the VPD54, VPD55, VPD56, and VPD57 mutants in LB-Tris/+EDDA/+Ent medium at pH 8.0. The growth assay was performed as described in Figure 1. Data are shown as means ± SD from 3 separate experiments.
