# Supplementary material for: Regulation of the Expression of the Vibrio parahaemolyticus peuA Gene Encoding an Alternative Ferric Enterobactin Receptor
Source: PLoS One. 2014 Aug 22;9(8):e105749. doi: 10.1371/journal.pone.0105749 (PMC4141801; doi:10.1371/journal.pone.0105749)
Supplement: Table S1 — Plasmids used in this study. (PDF) [file pone.0105749.s005.pdf]

Table S1. Plasmids used in this study

Plasmid	Description*	Reference or source
pXAC623	Suicide vector derived from pKTN701 containing the *sacB* gene of *B. subtilis*; Cm^r	[1]
pRK415	Broad-host-range plasmid (~10.5 kb); Tc^r	[2]
pXACΔpeuA	pXAC623 containing a 1,315-bp *Xho*I/*Sal*I-*Xba*I fragment with a 1,646-bp deletion in *peuA*; Cm^r	This study
pXACΔpeuRS	pXAC623 containing a 1,391-bp *Xba*I-*Xba*I fragment with a 1,654-bp deletion in *peuA*; Cm^r	This study
pXACΔtonB1	pXAC623 containing a 1,259-bp *Sac*I-*Sac*I fragment with a 507-bp deletion in *tonB1*; Cm^r	[3]
pXACΔtonB2	pXAC623 containing a 1,198-bp *Sac*I-*Sac*I fragment with a 504-bp deletion in *tonB2*; Cm^r	[3]
pXACΔtonB3	pXAC623 containing a 1,466-bp *Sac*I-*Sac*I fragment with a 511-bp deletion in *tonB3*; Cm^r	[3]
pXACΔpvuA1	pXAC623 containing a 1,151-bp *Xba*I-*Xho*I/*Sal*I fragment with a 1,657-bp deletion in *pvuA1*; Cm^r	[3]
pXACΔpvuA2	pXAC623 containing a 1,281-bp *Xba*I-*Xho*I/*Sal*I fragment with a 1,712-bp deletion in *pvuA2*; Cm^r	[3]
pXACΔhutA	pXAC623 containing a 1,713-bp *Kpn*I-*Kpn*I fragment with a 1,889-bp deletion in *hutA*; Cm^r	This study
pXACΔfhuA	pXAC623 containing a 2,252-bp *Xho*I/*Sal*I-*Xba*I fragment with a 1,221-bp deletion in *fhuA*; Cm^r	This study
pXACΔiutA	pXAC623 containing a 1,436-bp *Xba*I-*Xho*I/*Sal*I fragment with a 1,951-bp deletion in *iutA*; Cm^r	This study
pXACΔVP0168	pXAC623 containing a 1,107-bp *Xba*I-*Xba*I fragment with a 1,220-bp deletion in *VP0168*; Cm^r	This study
pRK415-peuA	pRK415 containing a 2,960-bp *Xba*I-*Xba*I fragment with full-length *peuA*; Tc^r	This study
pRK415-peuRS	pRK415 containing a 3,045-bp *Xba*I-*Xba*I fragment with full-length *peuRS*; Tc^r	This study

*Ap^r, ampicillin resistance; Cm^r, chloramphenicol resistance; Tc^r, tetracycline resistance

References

1. Kuroda T, Mizushima T, Tsuchiya T (2005) Physiological roles of three Na^+/H^+ antiporters in the halophilic bacterium *Vibrio parahaemolyticus*. Microbiol Immunol 49: 711-719.

2. Keen NT, Tamaki S, Kobayashi D, Trollinger D (1988) Improved broad-host-range plasmids for DNA cloning in gram-negative bacteria. Gene 70: 191-197.

3. Tanabe T, Funahashi T, Okajima N, Nakao H, Takeuchi Y et al. (2011) The *Vibrio parahaemolyticus pvuA1* gene (formerly termed *psuA*) encodes a second ferric vibrioferrin receptor that requires *tonB2*. FEMS Microbiol Lett 324: 73-79.
