# Supplementary material for: Regulation of the Expression of the Vibrio parahaemolyticus peuA Gene Encoding an Alternative Ferric Enterobactin Receptor
Source: PLoS One. 2014 Aug 22;9(8):e105749. doi: 10.1371/journal.pone.0105749 (PMC4141801; doi:10.1371/journal.pone.0105749)
Supplement: Table S2 — PCR primers used in this study. (PDF) [file pone.0105749.s006.pdf]

**Table S2.** PCR primers used in this study

| Purpose for | Primer name | Sequence (5′–3′)* |
|---|---|---|
| construction of Δ*peuRS* fragment | VppeuRS–1 (including *Xba*I site) | AATATCTCTAGAATCGATGCTGATGAGTTGC |
| | VppeuRS–2 | agacagacatgcaacatcacATCGAGCAAC |
| | VppeuRS–3 | gtgatgttgcatgtctgtctCTGACGATGG |
| | VppeuRS–4 (including *Xba*I site) | CACGTATCTAGAAACGTTCACGGTTACGG |
| confirmation of *peuRS* deletion | VppeuRS–5 | CATTGTTGCTAACCGTGTGG |
| | VppeuRS–6 | TGTGTTTGCGTTGTTGGTTT |
| construction of full-length *peuRS* | VppeuRS–1 | See above |
| | VppeuRS–4 | See above |
| construction of Δ*peuA* fragment | VppeuA–1 (including *Xho*I site) | TGACCGACTCGAGAGCTTACTGTGAAGATAC |
| | VppeuA–2 | gaccatagacgctgtactgtTGGTATCAGC |
| | VppeuA–3 | acagtacagcgtctatggtcTAGCTACAGG |
| | VppeuA–4 (including *Xba*I site) | CAAACTTTCTAGATGATCACGATAAATCTTC |
| confirmation of *peuA* deletion | VppeuA–5 | TCCTGTGGCATTTGTTGTGT |
| | VppeuA–6 | ATGGTCGCAATTTCGCTAAC |
| construction of full-length *peuA* | VppeuA–7 (including *Xba*I site) | TGACCGAGTCTAGAGCTTACTGTGAAGATAC |
| | VpryhB–4 | See above |
| construction of Δ*hutA* fragment | VphutA–1 (including *Kpn*I site) | TAGGTAAGGTACCAATCCCTAAGCCTG |
| | VphutA–2 | cacgcacatcattccaattgagtcacgaccacttcattgAAGG |
| | VphutA–3 | caatgaagtggtcgtgactcaattggaatgatgtgcgtgACC |
| | VphutA–4 (including *Kpn*I site) | CAACCTTGGTACCGGTAATGGTTACAG |
| confirmation of *hutA* deletion | VphutA–5 | CATTGTTGCTAACCGTGTGG |
| | VphutA–6 | TGTGTTTGCGTTGTTGGTTT |
| construction of Δ*fhuA* fragment | VpfhuA–1 (including *Xho*I site) | GATATACTCGAGTACATCCACCAGCTTCATC |
| | VpfhuA–2 | gtgatgtcgtcttgtgcgttCGCTGAAAGC |
| | VpfhuA–3 | aacgcacaagacgacatcacTTCACAGTGG |
| | VpfhuA–4 (including *Xba*I site) | GTAAAGTCTAGACAAACGCGCTTCATCAAG |
| confirmation of *fhuA* deletion | VpfhuA–5 | AAACCGTCACCAAAAACTGC |
| | VpfhuA–6 | CGAACCATCATGCACATACC |
| construction of Δ*iutA* fragment | VpiutA–1 (including *Xba*I site) | TTGGCTCTCTAGAACATGGGATTTGCTCTG |
| | VpiutA–2 | atcttcatcaccagttgctgAGCTTGTTCC |
| | VpiutA–3 | cagcaactggtgatgaagatTACACCACGG |
| | VpiutA–4 (including *Xho*I site) | GTAAGCGCTCGAGGTATCACGCTTCACTAAC |
| confirmation of *iutA* deletion | VpiutA–5 | CAAACATCGCAAACCATCAC |
| | VpiutA–6 | TTTCGCGTACTCGATCACTG |
| construction of Δ*VP0168* fragment | VP0168–1 (including *Xba*I site) | AGAATATCTAGACACATAAGCTGTGGTTAC |
| | VP0168–2 | tgagtttgccttctgcctgtGCTGTTAGAG |
| | VP0168–3 | acaggcagaaggcaaactcaGGTCAGTTTG |
| | VP0168–4 (including *Xba*I site) | TGAGTTTCTAGACATTCTGAAGCTGATATG |
| confirmation of *VP0168* deletion | VP0168–5 | TTCCTCCGATGTCGAAAAAC |
| | VP0168–6 | CATGCTCTGGAGCGTATTCA |

| | | |
|---|---|---|
| RT-PCR primers | VppeuR-F | CGCTGCAGTACATGGAGAAA |
| | VppeuS-R | AAGCGAGAACAAAGCCAAAA |
| | VppeuA-F | CGTTCAAACAGATGAGCACC |
| | VppeuA-R | AGAAACGTTCACGGTTACGG |
| | VppeuA-F2 | GAGTTGTCGCTAACCGCTTC |
| | VPA0151-R | ATGTTGGCTTTCGACCAAAC |
| | VPA0151-F | AAATGGACTACGGCACCAAG |
| | VpttpC2-R | TGAAAGAGACCTTCGCCAGT |
| | VpttpC2-F | GTGACAACGGTTCTCGGTTT |
| | VptonB2-R | TGACGCTGGTAACGCAATTA |
| | VpexbD2-F | GATCTGGAACGCGTAGAAGC |
| | VPA0156-R | GCGGTGTACTGGCTAAGCTC |
| primer extension for *peuR* | VppeuR-PE | GCAACATCACATCGAGCAAC |
| primer extension for *peuA* | VppeuA-PE | GGTGCTCATCTGTTTGAACG |
| RT-qPCR primers specific to *peuA* | VppeuA-qF | CGAACTACCTCGACTACAAATACG |
| | VppeuA-qR | CTACGTCCTCGCCTGCAT |
| RT-qPCR primers specific to 16S rRNA | q16S1 | GTTGGTGAGGTAAGGGCTCA |
| | q16S2 | GCTGATCATCCTCTCAGACCA |
| construction of template DNA for *in vitro* transcription of T7-*peuA-flag* 5′-UTR (long) | T7-VppeuA-F | AAAACTTAATACGACTCACTATAGGGAGATTAATGCAAATGGTAATACTTATC |
| | T7-VppeuA-FLAG-R | TATTCATTATTTATCGTCGTCATCTTTGTAGTCCGAGAAACGTTCACGGTTACGG |
| construction of template DNA for *in vitro* transcription of T7-*peuA-flag* 5′-UTR (short) | T7-VppeuA-F2 | ATGGTATAATACGACTCACTATAGGGAGAGCAAAATAAATAAGAGGGAATCAC |
| | T7-VppeuA-FLAG-R | See above |
| construction of template DNA for *in vitro* transcription of T7-*fur-flag* | T7-Vpfur-F | AAGGAGATATACCAATGTCAGATAATAATCAGGC |
| | T7-Vpfur-FLAG-R | TATTCATTATTTATCGTCGTCATCTTTGTAGTCTTTTGCAGGTTTGTGCGCATC |
| | UNIVERSAL | GAAATTAATACGACTCACTATAGGGAGACCACAACGGTTTCCCTCTAGAAATAATTTTGTTTAACTTTAAGAAGGAGATATACCA |
| construction of DIG-labeled *peuA* probe for northern blot | VppeuA-F | CGTTCAAACAGATGAGCACC |
| | VppeuA-R | AGAAACGTTCACGGTTACGG |

* Underlined sequences indicate restriction enzyme sites; lower-case letter sequences within primers #2 and #3 are complementary base pairs.
